# Morphological divergence of lake and stream *Phoxinus* of Northern Italy and the Danube basin based on geometric morphometric analysis

**DOI:** 10.1002/ece3.2648

**Published:** 2016-12-20

**Authors:** David Ramler, Anja Palandačić, Giovanni B. Delmastro, Josef Wanzenböck, Harald Ahnelt

**Affiliations:** ^1^ First Zoological DepartmentMuseum of Natural History ViennaViennaAustria; ^2^ Department of Limnology and Bio‐OceanographyUniversity of ViennaViennaAustria; ^3^ Carmagnola Natural History MuseumCarmagnolaItaly; ^4^ Research Institute for Limnology MondseeUniversity of InnsbruckMondseeAustria; ^5^ Department of Theoretical BiologyUniversity of ViennaViennaAustria

**Keywords:** body shape, Cyprinidae, freshwater fishes, general Procrustes analysis, geometric morphometrics, *Phoxinus lumaireul*, *Phoxinus phoxinus*

## Abstract

Minnows of the genus *Phoxinus* are promising candidates to investigate adaptive divergence, as they inhabit both still and running waters of a variety of altitudes and climatic zones in Europe. We used landmark‐based geometric morphometric methods to quantify the level of morphological variability in *Phoxinus* populations from streams and lakes of Northern Italy and the Danube basin. We analyzed body shape differences of populations in the dorsal, lateral, and ventral planes, using a large array of landmarks and semilandmarks. As the species identification of *Phoxinus* on morphological characters is ambiguous, we used two mitochondrial genes to determine the genetic background of the samples and to ensure we are comparing homogenous groups. We have found significant body shape differences between habitats: Minnow populations inhabiting streams had a deeper body and caudal peduncle and more laterally inserted pectoral fins than minnows inhabiting lakes. We have also found significant body shape differences between genetic groups: Italian minnows had deeper bodies, deeper and shorter caudal peduncles, and a shorter and wider gape than both groups from the Danube. Our results show that the morphology of *Phoxinus* is highly influenced by habitat and that body shape variation between habitats was within the same range as between genetic groups. These morphological differences are possibly linked to different modes of swimming and foraging in the respective habitats and are likely results of phenotypic plasticity. However, differences in shape and interlandmark distances between the groups suggest that some (though few) morphometric characters might be useful for separating *Phoxinus* species.

## 1 Introduction

Many fish species are able to thrive in different types of water bodies with varying environmental parameters (Ehlinger & Wilson, 1988; Webb, 1984). If exposed to different hydrodynamic conditions, conspecific populations show divergence with regard to their morphology, physiology, development, or behavior (Robinson & Wilson, 1995; Spoljaric & Reimchen, 2011; Walker, 1997). Fish species occurring in contrasting habitats, such as benthic versus pelagic zone (Kahilainen et al., 2011; McPhail, 1993; Præbel et al., 2013; Willacker, von Hippel, Wilton, & Walton, 2010), or in lakes versus streams (Berner, Grandchamp, & Hendry, 2009; Brinsmead & Fox, 2002; Sharpe, Räsänen, Berner, & Hendry, 2008), often exhibit adaptive differences. These particularly concern locomotion (Brinsmead & Fox, 2002; McGuigan, Franklin, Moritz, & Blows, 2003; Sharpe et al., 2008) and foraging morphology (Berner, Adams, Grandchamp, & Hendry, 2008; Kahilainen et al., 2011). Such habitat‐induced morphological divergence was found in three‐spine stickleback *Gasterosteus aculeatus* (Walker, 1997; Wootton, 2009 and citations therein) and mosquitofish *Gambusia* sp. (Langerhans, Gifford, & Everton, 2007; Langerhans & Reznick, 2010). Divergent phenotypic adaptations to different habitats aid in an optimal utilization of local resources (Robinson & Wilson, 1994) and can facilitate phenotypic and genetic differentiation of subpopulations finally leading to speciation (Pfennig et al., 2010; Schluter, 2001; Vega‐Trejo, Zúniga‐Vega, & Langerhans, 2014). The role of phenotypic plasticity in evolution is still under debate (Ghalambor et al., 2015; Price, Qvarnström, & Irwin, 2003). On the one hand, it is regarded as a constraint to speciation processes (Ancel, 2000; Stearns, 1982), while on the other hand, plasticity can also be adaptive and contribute to genetic differentiation and thus speciation (Adams & Huntingford, 2004; Agrawal, 2001). The separation of intraspecific plastic responses to the environment from genetic differentiation is not only of interest for evolutionary biology, but also for conservation biology, an issue of increasing importance (Jacquemin, Martin, & Pyron, 2013).

Minnows of the genus *Phoxinus* Rafinesque 1820 are promising candidates to investigate adaptive divergence, as they inhabit both still and running waters at a variety of altitudes and climatic zones in Europe (Banarescu, 1992; Frost, 1943; Kottelat & Freyhof, 2007). We focused on minnows of Northern Italy and the Danube basin, which currently belong to two species, *Phoxinus lumaireul* and *Phoxinus phoxinus*, respectively. However, recent morphological (Kottelat, 2007; Kottelat & Freyhof, 2007) and molecular studies (Geiger et al., 2014; Knebelsberger, Dunz, Neumann, & Geiger, 2015; Palandačić, Bravničar, Zupančič, Šanda, & Snoj, 2015) indicate that *P. phoxinus* is a species complex. In addition, there has been contrasting evidence regarding the validity of *P. lumaireul* (Bianco, 2014). In preliminary studies (Palandačić, Ramler, Bravničar, Snoj, & Ahnelt, 2015; Ramler, Delmastro, Palandacic, Ahnelt, & Mikschi, 2015), we found that the distinguishing features of the Italian minnow *P. lumaireul*, as described by Kottelat and Freyhof (2007), did not allow the clear separation from *P. phoxinus*. Because morphological species delimitation was found to be unreliable, we used mitochondrial DNA markers cytochrome *b* and cytochrome oxidase I (COI) to ensure that we compared genetically uniform populations. Even though species delimitation is not an aim of the current study (nor is testing the species validity), geometric morphometric methods have been shown to be powerful tools for detecting speciation processes (Kerschbaumer, Mitteroecker, & Sturmbauer, 2014; Kerschbaumer, Postl, Koch, Wiedl, & Sturmbauer, 2010; Ramler, Mitteroecker, Shama, Wegner, & Ahnelt, 2014). Thus, we report here also the findings, which could contribute to resolving taxonomical issues in the genus *Phoxinus* in future studies.

To summarize, our aims were to (1) assess morphological differentiation between genetically homogeneous minnow populations inhabiting lakes and streams; (2) assess morphological divergence between genetically different populations inhabiting similar habitats; and (3) compare the extent of morphological differences between habitats and genetic groups.

## 2 Material and Methods

### 2.1 Ethical statement

Fish samples were caught under the permission of the concerned state, federal, or private agencies and institutions and were in accordance with the Austrian and Italian state laws on fisheries (Austria: Wiener Fischereigesetz LGBl. Nr. 01/1948; Niederösterreichisches Fischereigesetz 2001, LGBl. 6550‐6; Steiermärkisches Fischereigesetz 2000, LGBl. Nr. 52/2014. Italy: L.R. 37/2006—Regolamento Regionale sulla pesca 2012 1/R). For Austria: River Wien—Regional Government of Vienna MA 22 (Amt der Wiener Landesregierung für Umweltschutz), Lake Grundlsee—Austrian Federal Forest Agency (Österreichische Bundesforste AG), Lake Lunz—Forestry Administration Kupelwieser (Forstverwaltung Kupelwieser). For Italy: City of Turin, Det. no. 372‐48543/2014 (Servizio Caccia e Pesca della Città Metropolitana di Torino). As our study did not include experiments on living organisms, no further permissions from federal animal welfare agencies or ethics commissions were required.

### 2.2 Sampling and preservation

**Figure 1:**
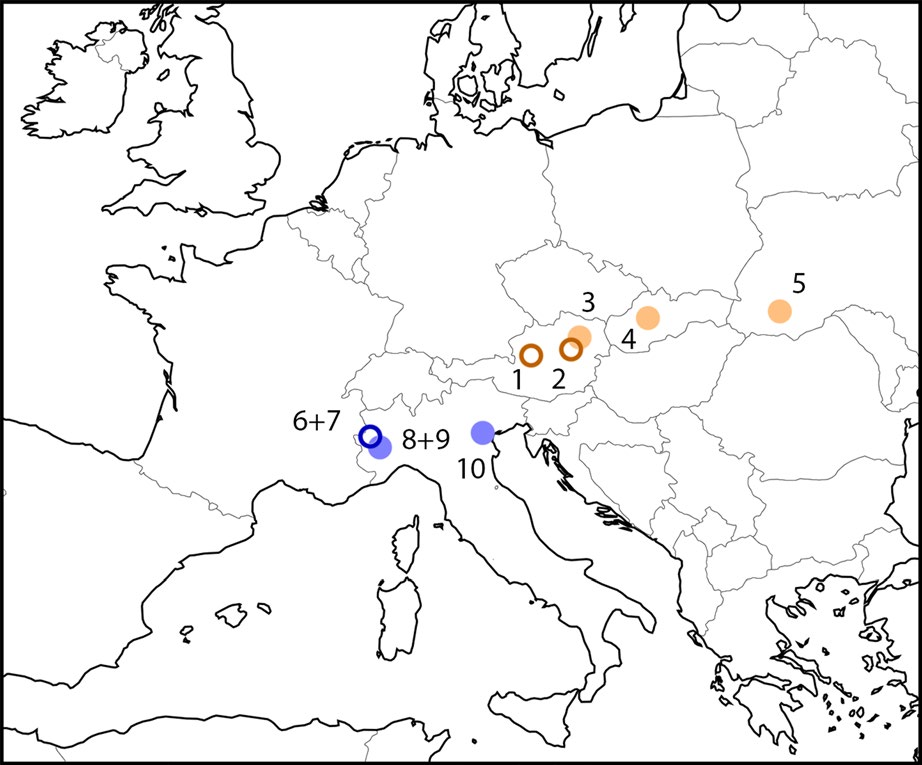
Location of sampling sites. Lakes indicated by circles; streams indicated by dots. 1, Lake Grundlsee (Goessl, AT); 2, Lake Lunz (Lunz am See, AT); 3, Wien River (Purkersdorf and Hütteldorf, AT); 4, Bystrica (Banska Bystrica, SK); 5, Pruth (tributary at Worochta, UA); 6, Lake Paradiso delle Rane (San Giorio di Susa, IT); 7, Lake Lau (Roreto Chisone, IT); 8, Chisone (Pinerolo, IT); 9, Sangone (Trana, IT); 10, Sile (Treviso, IT). For more information on the localities, see Table 1

We analyzed minnows from 10 European populations, from streams (S) and lakes (L) of Northern Italy (ITA) and the Danube basin (DAN; Figure 1). All captured minnows belong to the cyprinid genus *Phoxinus* and were divided into groups according to habitat and genetic background (see below). Characteristics of all localities and groupings are summarized in Table 1. Throughout the text, the term “lake” is used as a synonym for standing water bodies, while the term “stream” is used for flowing waters. Samples consisted of males and females. To account for possible effects of sexual dimorphism, all analyses were carried out for males and females separately.

**Table 1:** Genetic group, geographic origin of the populations used in this study (name of nearest village and water body), the registration number, habitat type, altitude, sample sizes of all planes for the geometric morphometric analysis, and year of sampling

Group	Locality	Water body	Country	NMW number	Habitat	Altitude	*n*_dorsal_	*n*_lateral_	*n*_ventral_	Year
DAN L	Goessl	Grundlsee	Austria	98654	Lake	708	30	30	30	2015
	Lunz am See	Lunzer See	Austria	51209, −216	Lake	608	17	17	17	1899
	Lunz am See	Lunzer See	Austria	98655	Lake	608	25	22	24	2014
DAN S	Worochta	Pruth	Ukraine	51238, –254, –260, –265, –269, –272, –286	Stream	748	24	15	23	1900
	Banská Bystrica	Bystrica	Slovakia	51246, –279, –288, –291	Stream	362	22	22	22	1900
	Hütteldorf/Purkersdorf	Wienfluss	Austria	98664	Stream	203	25	25	24	2014
ITA L	Roreto Chisone	L. del Lau	Italy	98658	Lake	2271	30	30	24	2014
	S. Gioro di Susa	L. Paradiso delle Rane	Italy	98660	Lake	1235	30	30	29	2014
ITA S	Trana	Sangone	Italy	98661	Stream	395	30	30	30	2014
	Pinerolo	Chisone	Italy	98662	Stream	340	30	30	29	2014
	Treviso	Sile	Italy	51268	Stream	15	11	11	0	1844
						Σ	274	262	252	

NMW, Museum of Natural History Vienna.

All specimens are stored at the Fish Collection of the Museum of Natural History in Vienna (NMW) and include already existing material complemented by recently caught fish (Table 1). From the large collection of *Phoxinus* samples at the NMW, we selected only those that were in excellent condition, comparable to recently caught ones. The fish sampled in 2014 and 2015 were caught by electrofishing or beach seine and were anaesthetized and then killed with an overdose of MS‐222 (Sigma‐Aldrich Co., St. Louis, MO, USA), to minimize suffering. Subsequently, the specimens went through an ascending alcohol series and were ultimately preserved in 75% alcohol like all museum specimens. Because preservation leads to shrinkage and weight loss of the specimens, which could affect morphometry (Buchheister & Wilson, 2005; König & Borcherding, 2012; Thorstad et al., 2007), all specimens were stored in 75% alcohol for at least two months prior to scanning, to account for effects of shrinkage. Several studies have shown that the shrinkage follows an exponential function and remains virtually stable after an initial phase of approximately one month (König & Borcherding, 2012; Kristoffersen & Salvanes, 1998; Moku, Mori, & Watanabe, 2004).

### 2.3 Genetic analysis

According to Kottelat and Freyhof (2007), the Italian populations should be assigned to *P. lumaireul* and all other examined populations to *P. phoxinus*. However, because of the taxonomic ambiguity within the genus *Phoxinus*, and uncertainty regarding morphological characteristics for species determination (see Introduction), we used mitochondrial DNA markers to ensure that we were comparing homogenous groups. Thus, three to five specimens from each population were genetically characterized for two genes, cytochrome *b* (cyt *b*) and COI. DNA was extracted from fin tissue using DNeasy Blood & Tissue Kit (Qiagen) following the manufacturer's protocol. Polymerase chain reaction (PCR) was performed with primers GluF (5′‐AACCACCGTTGTATTCAACTACAA‐3′) and ThrR (5′‐ACCTCCGATCTTCGGATTACAAGACCG‐3′) for cyt *b* (Zardoya & Doadrio, 1999), and FishF1 (5′‐TCAACCAACCACAAAGACATTGGCAC‐3′) and FishR1 (5′‐TAGACTTCTGGGTGGCCAAAGAATCA‐3′) for the COI (Ward, Zemlak, Innes, Last, & Hebert, 2005). Reaction volume was 25 μl, with 2.5 μl buffer, 500 μmol/L dNTPs, 1.5 μl (1.5 μmol/L) Mg2+, 0.25 μl of each primer (25pmol/μl), 0.15 μl TopTaq‐polymerase (0.5 units), and 2 μl DNA (with approx. concentration of 10 ng/μl). Cycling conditions for cyt *b* were as follows: initial denaturation 94°C 3 min, followed by 35 cycles of initial denaturation at 94°C for 30 s, annealing at 51°C for 30 s, and extension at 72°C for 60 s. The final extension was at 72°C for 10 min. For COI, annealing was set to 55°C and extension only lasted 45 s. Purification and sequencing (in both directions) of PCR products was performed by LGC Genomics (Berlin, Germany), with primers used for PCR. The sequences were edited by eye and aligned with MEGA 5.0 (Tamura et al., 2011). The same program was used for calculation of genetic distances between the detected genetic groups. The museum material used in this study was also genetically characterized, but because museum DNA is typically fragmented, only parts of cyt *b* were sequenced and used for comparison with the recent material. Fragments of cyt *b*, of a length between 250 to 350 bp, were amplified using in‐house primers and in‐house protocols, in accordance with standard procedures for museum DNA (e.g., clean room, extraction, and negative controls, etc.). More details on DNA extraction, PCR conditions, and sequencing are available on request from the authors.

### 2.4 Group formation

For the genetic and morphological analysis, we compared intergroup and intragroup variations in order to justify the formation of four groups, which were then used for the geometric morphometric and statistical analyses.

### 2.5 Geometric morphometric and statistical analyses

**Figure 2:**
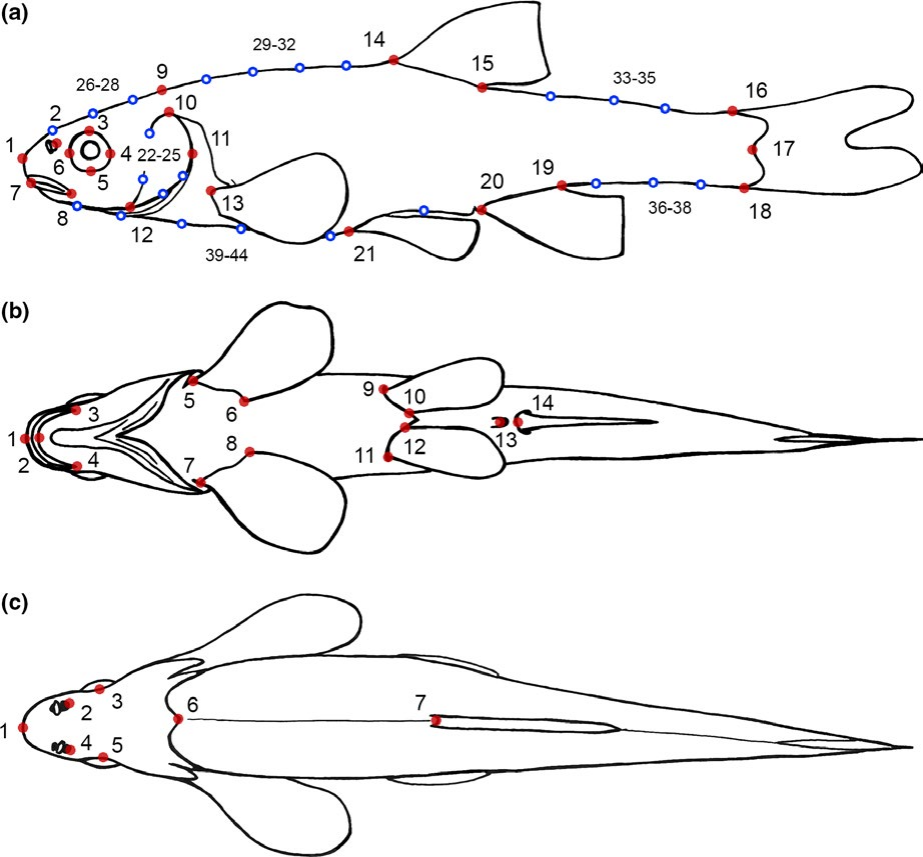
Sets of landmarks and semilandmarks used for geometric morphometric analysis and calculation of interlandmark distances. Landmarks indicated by red dots; semilandmarks indicated by blue circles (only lateral). (a) lateral side: (1) tip of snout, (2) posterior margin of posterior nare, (3–6) most dorsal, posterior, ventral, and anterior point of orbit, (7) opening of mouth, (8) posterior end of jaw, (9) posteriomedial tip of supraoccipital, (10–12) most dorsal, posterior, and ventral point of operculum, (13) origin of pectoral fin, (14, 15) origin and insertion of dorsal fin, (16) dorsal origin of caudal fin, (17) end of vertebral column, (18) ventral origin of caudal fin, (19, 20) insertion and origin of anal fin, (21) origin of pelvic fin, (22–25) outline of operculum, (26–28) outline of forehead, (29–32) predorsal outline of back, (33–35) postdorsal outline of back, (36–44) ventral outline. (b) ventral side: (1) tip of snout, (2) symphysis of lower jaw, (3, 4) left and right posterior edge of lip, (5, 6) origin and insertion of left pectoral fin, (7, 8) origin and insertion of right pectoral fin, (9, 10) origin and insertion of left pelvic fin, (11, 12) origin and insertion of right pelvic fin, (13) vent (centered on opening), (14) origin of anal fin. (c) dorsal side: (1) tip of snout, (2, 3) posterior margin of right and left posterior nare, (4, 5) most medial point of dorsal rim of right and left orbit, (6) posteriomedial tip of supraoccipital, (7) origin of dorsal fin

All fish were scanned on a flatbed scanner from the dorsal, (left) lateral, and ventral sides, following Herler, Lipej, and Makovec (2007). The sample sizes for each plane are given in Table 1. After randomization of the images, an extensive array of landmarks, based on Armbruster (2012), was digitized on every scan (Figure 2), using tpsDig2 (Rohlf, 2010a). We also followed Armbruster (2012) regarding the terminology of landmarks and the distances between them. On the lateral side, we also used semilandmarks to get information on curves and outlines where the homology criterion of classic landmarks cannot be met (Gunz & Mitteroecker, 2013). In total, we have used 21 landmarks (+23 semilandmarks) on the lateral side, 14 on the ventral side, and seven on the dorsal side. As the dorsal and ventral sides are not as flat as the lateral side, specimens were tilted cranially, to ensure that the front part of the fish is planar and as close as possible to the scanning glass. As a result, the more caudal body parts could not be used for the analysis and landmarks behind the insertion of the dorsal fin (dorsal side) or anal fin (ventral side) were omitted. A generalized Procrustes analysis (GPA) implemented in tpsRelw (Rohlf, 2010b) was used to standardize the landmark configurations for position, orientation, and size (Mitteroecker & Gunz, 2009; Rohlf & Slice, 1990). The resulting shape differences were illustrated by thin‐plate‐spline deformation grids (Bookstein, 1989). We used between‐group PCAs (bgPCA; Mitteroecker & Bookstein, 2011) to analyze and display the coordinates obtained from the GPA (Procrustes shape coordinates), which separated the groups better than a standard PCA. In cases where PCA revealed that principal components (PCs) were influenced by preservation artefacts (i.e., no actual variation in body shape), the respective PCs were removed from the data, by projecting the shape coordinates of the specimens into the subspace perpendicular to the PC (Burnaby, 1966). These artefacts are reflected in lunate or S‐shaped bendings and result from shrinkage during preservation or nonplanar storage (Kristoffersen & Salvanes, 1998; Valentin, Penin, Chanut, Sévigny, & Rohlf, 2008) and are repeatedly found in geometric morphometric studies on fishes (Cavalcanti, Monteiro, & Lopes, 1999; Leinonen, Cano, & Merilä, 2011; Sharpe et al., 2008). All further shape analyses, as well as the numbering of the reported PCs, were based on these residual data. The total variance, which is the trace of the corresponding covariance matrix, was used as a measure of shape variation and describes how morphometrically homogenous populations are. To assess statistical significances of group mean differences, we performed Monte Carlo permutation tests (10,000 permutations each), using Procrustes distance (PD) as the test statistic (Good, 2000). Level of significance was determined at α = 5%. Statistical significances were Bonferroni‐corrected, by multiplying the obtained *p*‐values by the number of tests performed. Allometric effects were estimated using a multivariate, pooled, within‐group regression of shape on centroid size (Klingenberg, 1998; Mitteroecker, Gunz, Windhager, & Schaefer, 2013). To assess the strength of the group separations, we conducted a multivariate discriminant analysis (DA), using the relative warp scores of the first five PCs (after removing PCs with bending artefacts) of each specimen as predicator variables. The accuracy of the classification was evaluated by leave‐one‐out cross‐validation. The DA was conducted with SPSS Statistics 20 (SPSS, Chicago, IL, USA), and all other morphometric and statistical analyses were conducted with Mathematica 8 (Wolfram Research Inc., Champaign, IL, USA).

In addition, we calculated interlandmark distances (Tables S1, S2) to look for traits which may be used as distinguishing features between habitats and genetic groups. All traits were standardized for standard length. Traits on the head or caudal peduncle were also standardized for head length or caudal peduncle length, respectively. We checked whether the respective groups show overlaps of the mean value of each trait ±1 *SD*, and also ±0.5 *SD*, as an estimate of the usefulness or reliability of a trait for group delimitation. We refrained from producing *p‐*values, as a statistically detectable significant difference of a trait among groups does not imply that the concerned trait is indeed useful as a distinguishing feature.

## 3 Results

### 3.1 Effects of size, sex, and preservation

Body size of individual fish varied among the populations, with an average length of 53.0 ± 7.8 mm (mean ± standard deviation). According to different studies (Frost, 1943; Mills, 1987; Mills & Eloranta, 1985), the size of maturity in European minnows is around 40–50 mm; thus, the investigated fish were adults. Allometry (i.e., shape differences related to size) had a significant (*p *<* *.001) effect on shape, but explained only 1.52% of the total variation in the data (measured only lateral). Apart from slightly larger eyes of smaller individuals, no major differences were apparent. Due to the very small fraction of explained variance, allometric effects were not further considered in the study.

All body shape differences between habitats and genetic groups were equally expressed in both males and females. The exception is a slightly larger ventral region of females compared with males, which in turn leads to a deeper body. However, body shape changes between habitats or genetic groups that concern the ventral region or body depth (BD) were also expressed when only males were examined. All other traits (e.g., caudal peduncle depth [CPD], head size, length of fin bases) were unaffected by sexual dimorphism. As a consequence, males and females were pooled for all analyses.

The PCA revealed that some of the PCs reflected preservation artefacts (bendings) without any actual variation in body shape. As a consequence, PCs 1 and 5 of the lateral data set, PCs 2 and 6 of the dorsal data, as well as PC 2 of the ventral data were removed, as they all depicted lunate‐like or S‐shaped bendings, but no shape changes that could be attributed to effects of habitat or genetic group (e.g., changes in body proportions).

### 3.2 Genetic analysis and group formation

Genetic analysis revealed three genetic groups, consisting of (1) the Danube lake populations (Grundlsee, Lunzer See), (2) the Danube stream populations (Pruth, Bystrica, Wienfluss), and (3) all Italian populations. The genetic distances between the groups are given in Table 2.

**Table 2:** Number of base substitutions per site from averaging over all sequence pairs between groups

	DAN L	DAN S	ITA S	ITA L
cyt *b*				
DAN L		0.01	0.01	0.01
DAN S	0.09		0.01	0.01
ITA S	0.06	0.08		0
ITA L	0.06	0.08	0.01	
COI				
DAN L		0.01	0.01	0.01
DAN S	0.05		0.01	0.01
ITA S	0.02	0.05		0
ITA L	0.02	0.05	0.01	

Standard error estimate(s) are shown above the diagonal and were obtained by a bootstrap procedure (1,000 replicates). Analyses were conducted using the Tamura–Nei model (Tamura & Nei, 1993), calculated to be the most appropriate model for the dataset. The differences in the composition bias among sequences were considered in evolutionary comparisons (Tamura & Kumar, 2002). The analysis involved 33 (cytochrome *b*; cyt *b*) and 35 (cytochrome oxidase I; COI) nucleotide sequences with a total of 589 (cyt *b*) and 651 (COI) positions. Evolutionary analyses were conducted in MEGA5 (Tamura, Dudley, Nei, & Kumar, 2007). DAN L, Danube lake populations; DAN S, Danube stream pop.; ITA L, Italian lake pop.; ITA S, Italian stream pop.

For the geometric morphometric analyses, we assigned the populations to four groups (Danube lake populations, DAN L; Danube stream pop., DAN S; Italian lake pop., ITA L; Italian stream pop., ITA S) to compare genetically identical groups from different environments and genetically different groups from the same environment. As DAN L and DAN S were genetically distant, we only used ITA L and ITA S for the habitat comparisons, to ensure that differences were not attributable to genetic differences.

To justify the groupings from a morphological point of view, we looked at each population individually (e.g., deformation grids, variances, PDs, PCAs) and generally found that intragroup variation was smaller than intergroup variation and that populations from the same habitat and genetic group share similar body shapes.

### 3.3 Geometric morphometrics

**Figure 3:**
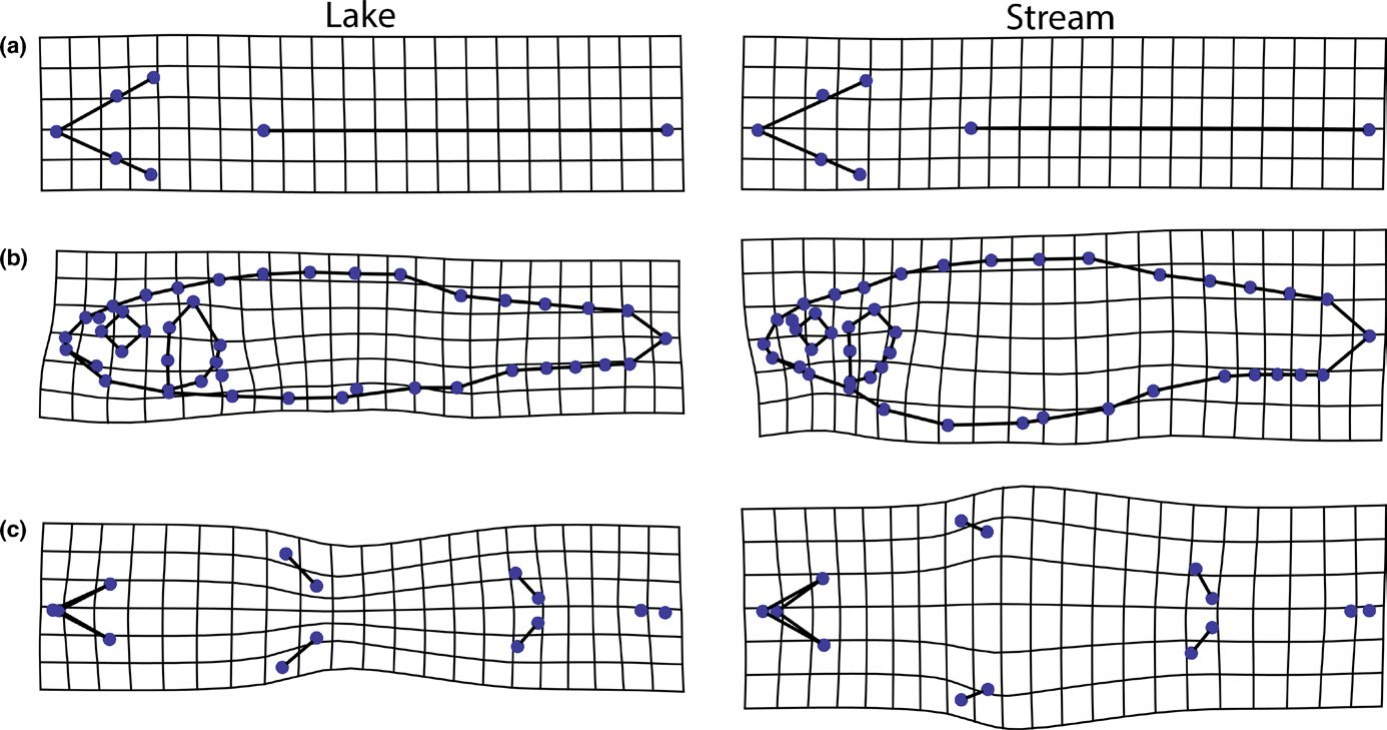
Shape differences between lake and stream populations in Northern Italy. Linearly extrapolated by factor 2. ITA L, lake populations; ITA S, stream populations. (a) dorsal; (b) lateral; (c) ventral

Significant differences in shape were found between ITA L and ITA S populations for the lateral and ventral sides (*p *<* *.001), but not for the dorsal side (*p *=* *.191 before Bonferroni correction; Figure 3; Table 3). Regarding the lateral side, ITA L had more slender bodies and caudal peduncles, shorter bases of the dorsal and anal fins, as well as larger heads and eyes. The larger head is primarily due to the increase in the eye, as both snout and postorbital head length remained unaffected. The mouth, terminal in ITA L, was slightly subterminal in ITA S minnows (Figure 3b). On the ventral side, ITA L had a distinctly narrower body and gape. The pectoral fins were closer together and also more vertically positioned. Furthermore, the bases of the pectoral fins were longer (Figure 3c).

**Table 3:** Total shape variances, Procrustes distances, and statistical significances for stream and lake populations in Northern Italy and the Danube basin

Group	dorsal	Lateral	Ventral
Shape variance			
ITA S	4.71	8.93	16.03
ITA L	5.46	8.30	17.60
DAN S	6.79	10.43	14.13
DAN L	7.08	9.76	15.43
PD			
ITA S versus ITA L	4.84	21.26^a^	35.81^a^
ITA S versus DAN S	14.72^a^	24.01^a^	37.98^a^
ITA L versus DAN L	17.43^a^	17.13^a^	35.29^a^

PD, Procrustes distance; S, stream populations; L, lake populations; ITA, Northern Italy; DAN, Danube basin. Statistical significances were estimated by Monte Carlo permutation tests (10,000 permutations each), using Procrustes distance as the test statistic.

a *p *<* *.001. See text for further information on group affiliations.

**Figure 4:**
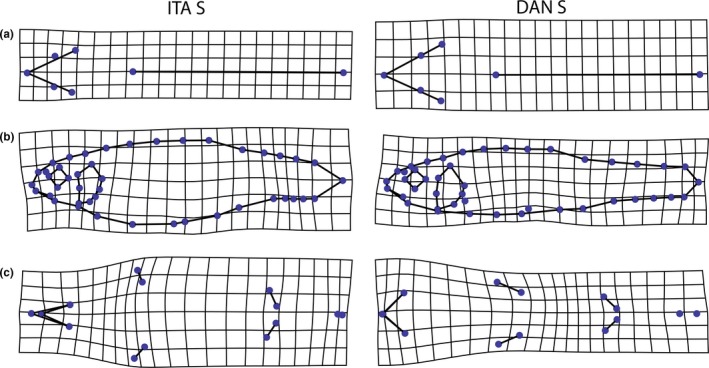
Shape differences between stream populations in Northern Italy and the Danube basin. Linearly extrapolated by factor 2. ITA S, stream populations in Northern Italy; DAN S, stream populations of the Danube basin. (a) dorsal; (b) lateral; (c) ventral

Highly significant shape differences were found between ITA S and DAN S for all body planes (all *p *<* *.001; Figure 4; Table 3). On the lateral side, DAN S minnows had more slender bodies and also more slender and longer caudal peduncles (Figure 4b). Ventrally, DAN S minnows had a distinctly shorter, but wider, gape and a more slender body. The bases of the pectoral fins were thus closer together. In addition, the base of pectoral fin was longer and more horizontally positioned. The distance between the pectoral and the pelvic fins was shorter; however, the distance between the anus and the anal fin was longer (Figure 4c). Minor differences were found on the dorsal side. DAN S samples exhibited a slightly longer and broader head and a shorter head to dorsal fin distance (Figure 4a).

**Figure 5:**
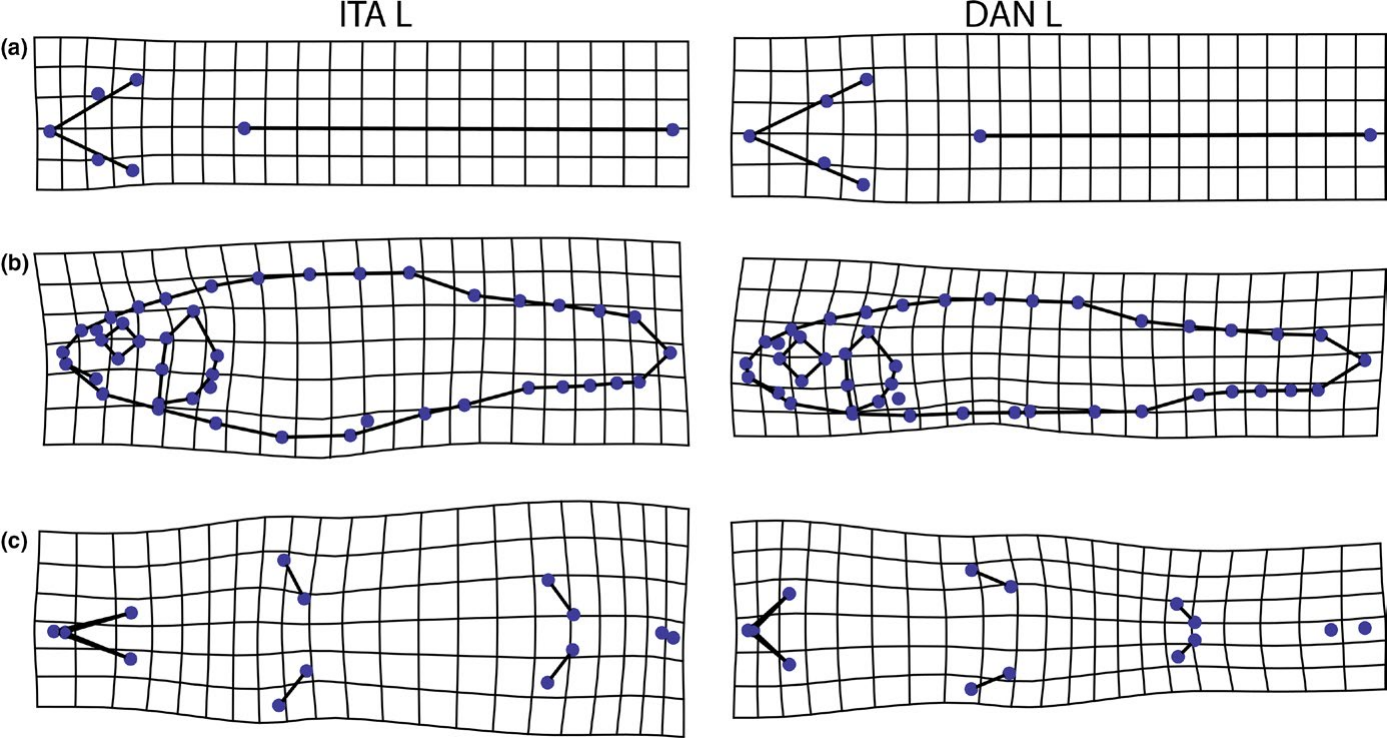
Shape differences between lake populations in Northern Italy and the Danube basin. Linearly extrapolated by factor 2. ITA L, lake populations in Northern Italy; DAN L, lake populations of the Danube basin. (a) dorsal; (b) lateral; (c) ventral

Highly significant shape differences were found between ITA L and DAN L for all body planes (all *p *<* *.001; Figure 5; Table 3). On the lateral side, DAN L had more slender bodies and also more slender and longer caudal peduncles. However, DAN L also exhibited bigger eyes (Figure 5b). Ventrally, DAN L minnows had a distinctly shorter and broader gape, but a more slender body. However, the bases of the pectoral fins were further apart and of similar relative length than of ITA L. The pelvic fins of DAN L were closer together and their bases shorter. The distance between the pectoral and the pelvic fins was shorter, but the distance between the anus and the anal fin was longer (Figure 5c). The minor differences on the dorsal side were similar to the changes between stream populations, including a slightly longer and broader gape. The distance from the nape to the dorsal fin was shorter in DAN L (Figure 5a).

A tabular summary of the main shape differences among the groups is provided in the Table S3.

**Figure 6:**
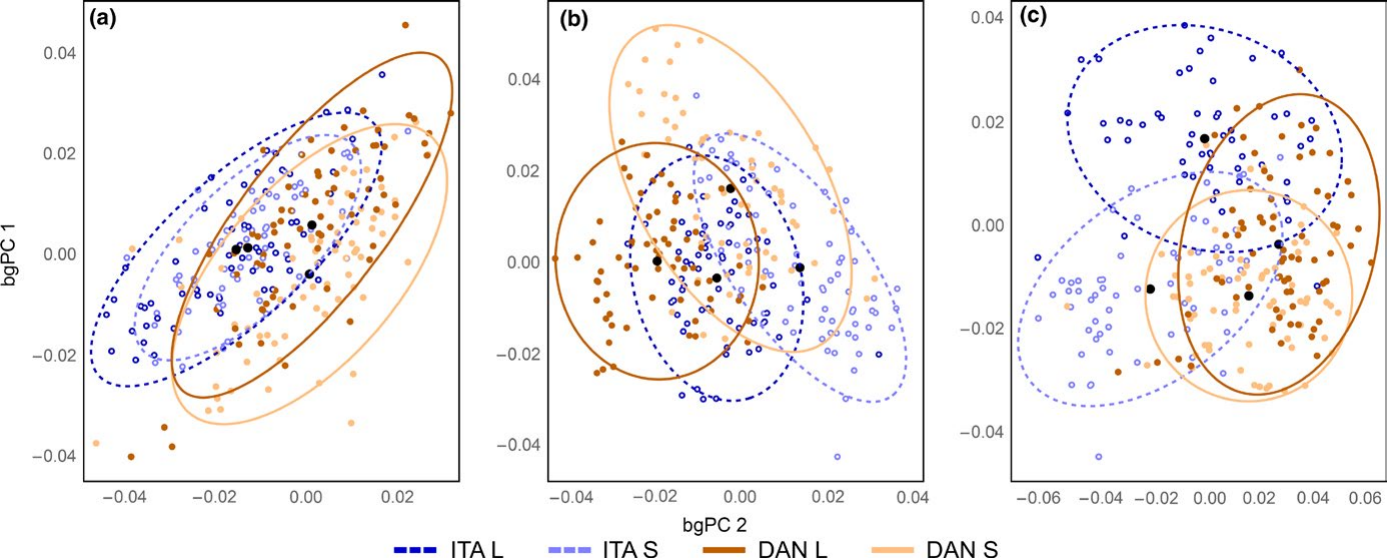
Between‐group principal component analysis of minnow body shape. Large black dots indicate mean shapes and the ellipses the 90% equal frequency ellipses for stream (bright) and lake (dark) populations in Northern Italy (ITA; dashed blue ellipses, circles) and the Danube basin (DAN; solid orange ellipses, dots). (a) dorsal; (b) lateral; (c) ventral

The dorsal plane showed the smallest values of shape variance and also small PDs indicating more similar shapes (Table 3). This is further reflected in the between‐group PC plot, as well as the DA in which the dorsal plane was generally the least suitable plane to distinguish between the groups. The ventral plane had the highest values for both shape variance and PD. Additionally, the DA also showed the highest percentage of correct classifications to the respective groups in most cases for the ventral plane, especially for the Italian populations. The lateral plane had intermediate values of shape variance, PD, and correct assignments to groups in the DA, and correct assignments to habitat group were the highest for this plane (Figure 6; Tables 3 and 4).

**Table 4:** Percentages of correct classifications of a multivariate discriminant analysis of the relative warp scores of the first five principal components of each plane, assessed by leave‐one‐out cross‐validation

Group	Dorsal	Lateral	Ventral
Population			
ITA S	43.7	70.4	86.4
ITA L	41.7	51.7	84.9
DAN S	62.0	53.2	58.0
DAN L	47.2	68.1	60.6
Total	48.9	61.5	71.0
Wilk's lambda	0.499	0.159	0.304
Genetic			
ITA S + ITA L	87.0	74.0	95.5
Group			
DAN S	57.7	48.4	55.1
DAN L	36.1	68.1	60.6
Total	66.1	66.4	74.6
Wilk's lambda	0.520	0.283	0.472
Habitat			
Lake	60.6	83.7	81.5
Stream	59.9	85.0	82.0
Total	60.2	84.4	81.7
Wilk's lambda	0.915	0.575	0.491
Region			
DAN	77.6	72.5	89.3
ITA	79.4	66.4	93.8
Total	78.5	69.5	91.3
Wilk's lambda	0.591	0.332	0.715

Wilk's lambda is given in the last line of each group (all discriminant functions were highly significant with *p *<* *.001). ITA, Northern Italy; DAN, Danube basin; S, stream populations; L, lake populations. See Table 1 and text for further information on group affiliations.

On the basis of means ±1 *SD*, the interlandmark distances showed overlaps in all traits and all comparisons. The only exception was the depth of the caudal peduncle (CPD), which had no overlap between DAN L versus ITA L. On the basis of means ±0.5 *SD*, there were still overlaps in all traits between the habitat groups (ITA S vs. ITA L). However, the number of traits without overlap increased for DAN L versus ITA L, including predorsal length, prepelvic length, CPD, and BD. Caudal peduncle depth also showed no overlap between DAN S versus ITA S (see Tables S2 and S3 for all distances).

## 4 Discussion

We analyzed body shape differences of populations of the genus *Phoxinus* in the dorsal, lateral, and ventral planes, using a large array of landmarks and semilandmarks. Previous studies mostly focussed on only one side (usually lateral) and used a small standard set of landmarks (Armbruster, 2012; Brinsmead & Fox, 2002; Simonović, Garner, Eastwood, Kováč, & Copp, 1999). The inclusion of sliding landmarks and all planes allowed a thorough examination of body shape changes and, to some extent, also a cross‐validation of the results (e.g., if the same patterns are found on different planes). We found the lowest values for shape variance on the dorsal side, and, furthermore, the DA showed low percentages of correct classifications on this side. The shape changes were mainly attributed to ventral body regions, again supported by the DA. Thus, besides using the (popular) lateral side, we strongly suggest using also the ventral side in similar future studies. The bgPCA and PDs showed that differences in body shape between habitats and genetic groups varied, but was generally within the same range, pointing to strong environmental effects on body shape.

### 4.1 Body shape differences between habitats (ITA L vs. ITA S)

The present study revealed a deeper body and caudal peduncle, as well as more laterally inserted pectoral fins of stream minnows in contrast to a more streamlined (i.e., more slender) body in lake minnows. Even though it is seemingly paradoxical that stream minnows exhibit a less streamlined body, our findings are in accordance with their habitat preferences. In streams, minnows prefer habitats characterized by slow flowing water, for example, close to the shore (Frost, 1943; Garner, Clough, Griffiths, Deans, & Ibbotson, 1998; Simonović et al., 1999; Tack, 1940). Thus, instead of flow velocity, habitat complexity of river banks, which favors high maneuverability attained through a deeper body and laterally positioned pectoral fins, might be the driving force behind differences in body shape of the stream minnow populations detected in this study. Complex habitats of stream banks favor high maneuverability, attained by fish through a deeper body, laterally positioned pectoral fins and enlarged dorsal and anal fins (Ehlinger & Wilson, 1988; Gerstner, 1999; Robinson & Wilson, 1995; Webb, 1984). In contrast, large open water bodies with patchily distributed food favor steady and sustained swimming and thus a more streamlined body with pectoral fins placed more medially (Ehlinger & Wilson, 1988; Langerhans & Reznick, 2010; Walker, 1997; Webb, 1984). The only sexual dimorphic character found was body depth, due to a slightly larger abdomen of the females. However, all body shape differences described above were also present when only males were examined. The general changes in body shape are thus not affected by different sex ratios, or different extents of sexual dimorphism among the populations.

Lake minnows in our study had larger heads (Figure 3), which might be due to habitat‐induced changes in head structures linked to different modes of foraging (Ahnelt, Keckeis, & Mwebaza‐Ndawula, 2015; Eklöv & Jonsson, 2007; Langerhans, Layman, Langerhans, & Dewitt, 2003; Svanbäck & Eklöv, 2003). However, the head is larger owing to larger eyes, which have been shown to increase the visual acuity (Land & Nilsson, 2002; Wanzenböck, Zaunreiter, Wahl, & Noakes, 1996), both in feeding (Berner et al., 2008) and predator avoidance (Goatley, Bellwood, & Bellwood, 2010). The lake minnows in this study were also characterized by a narrower gape. A decrease in gape width supposedly corresponds to higher foraging success in open water, while a larger gape is favorable in feeding on benthic macro‐invertebrates (Svanbäck & Schluter, 2012; Walker, 1997). Studies dealing with the diet of minnows found that lake minnows feed primarily on small prey, while stream minnows prefer larger food (Frost, 1943; Michel & Oberdorff, 1995; Straskraba, Chiar, Frank, & Hruska, 1966; Tack, 1940). Consequently, we suggest that our findings reflect divergent adaptations to the respective habitat types in both lake and stream minnows, intertwined with trophic niche partitioning, as have been found for various families, such as Gasterosteidae (Berner et al., 2008; Hendry & Taylor, 2004; Walker, 1997), Cyprinidae (Jacquemin et al., 2013), or Poeciliidae (Langerhans et al., 2007; Robinson & Wilson, 1995). Nevertheless, Collin and Fumagalli (2011), who examined minnows in Switzerland, found deeper bodies and caudal peduncles in lake populations, that is, the opposite to our findings. These authors attributed shape differences between lake and stream minnows to high predatory pressure by salmonid fishes, implying that the shape changes induced by predatory pressure can overcome those of sustained swimming in open water. Predatory pressure in open waters might also result in a deeper posterior body, which can enhance rapid acceleration (Langerhans et al., 2003, 2007; Spoljaric & Reimchen, 2007; Walker, 1997).

### 4.2 Body shape differences between genetic groups (ITA S vs. DAN S; ITA L vs. DAN L)

By comparing genetically divergent groups from the same habitat, we aimed to identify morphological changes in body shape which are unaffected by the environment. We found differences in overall body shape between Italian and Danube populations, with Italian minnows having deeper bodies and deeper and shorter caudal peduncles in both habitat types. Additionally, all Italian populations had longer jaws, distinctly narrower gapes and snouts, pectoral fin bases originating in a steep angle, and a shorter distance between the anus and origin of the anal fin (Figures 4 and 5). Interestingly, Collin and Fumagalli (2015) found similar differences in body shape between Alpine minnows (elongate body) and minnows from the Pyrenees (deep body). These identified differences might be a good orientation for morphological species assignment. In addition to differences in body shape, our study indicates that Italian minnows might be differentiated from Danube minnows using genetic methods, thus supporting the reestablishment of *P. lumaireul* in the Po tributary by Kottelat (2007). Because we only used mitochondrial genes and further molecular studies are needed for phylogenetic and/or taxonomic inference, here we only report trends noticed based on comparisons of genetic distances. As cited in the section (1) of the discussion, body shape differences can also be attributed to environmental factors (i.e., predation) not accounted for in this study. To ensure whether the morphological differences are due to genetic or rather ecological differences (or a mixture of both) would require a common garden experiment. Nevertheless, the changes which we detected might aid further taxonomical studies to find distinguishable characters between the *Phoxinus* species.

### 4.3 Comparison of body shape differences between habitats (L vs. S) and genetic groups (ITA vs. DAN)

The results of the DA and the detected shape differences suggest that the extent of morphological differences between genetic groups is within the same range as between different environments (Figures 4, 5, 6; Table 4). Furthermore, the shape changes we found between ITA L and ITA S closely resemble the ones between DAN L and DAN S, although considerable genetic distance separates DAN L from DAN S. Thus, despite the genetic distance of 9% based on cyt *b* and 5% based on COI (Table 2), the morphological differences detected between DAN L and DAN S appear to be a consequence of habitat. It is conceivable that, in extreme cases, habitat‐induced body shape changes (e.g., head length, eye diameter, gape and body width) may mask morphological differences between species/genetic groups (Langerhans & Reznick, 2010; Langerhans et al., 2007; Lucek, Kristjánsson, Skúlason, & Seehausen, 2016; Walker, 1997). This should be accounted for in future approaches on morphological species delimitations in this genus. Further sampling and analysis of genetically homogenous groups from mixed habitats of the Danube basin should be performed in order to draw any final conclusions.

However, some morphometric characters might be useful for separating *Phoxinus* species. The few traits we have found, which may be used as distinguishing features, concern either the depth of the body or caudal peduncle, or the insertion of fins. In particular, caudal peduncle depth appears to be a good trait for separating Italian populations from the Danube populations. Nevertheless, all traits generally showed some extent of overlap and may thus only be useful in combination.

## Conflict of Interest

None declared.

## Data accessibility

Raw landmark coordinates, used in the geometric morphometric analysis, are provided in the Supporting Information. All genetic sequences are deposited in GenBank under accession numbers KX673409–KX673485.

## References

[ece32648-bib-0001] Adams, C. E. , & Huntingford, F. A. (2004). Incipient speciation driven by phenotypic plasticity? Evidence from sympatric populations of Arctic charr. Biological Journal of the Linnean Society, 81, 611–618.

[ece32648-bib-0002] Agrawal, A. A. (2001). Phenotypic plasticity in the interactions and evolution of species. Science, 294, 321–326. 11598291 10.1126/science.1060701

[ece32648-bib-0003] Ahnelt, H. , Keckeis, H. , & Mwebaza‐Ndawula, L. (2015). Rapid phenotypic divergence in the small African cyprinid *Rastrineobola argentea* (Pellegrin 1904) (Teleostei: Cyprinidae) in Lake Victoria, Uganda. African Journal of Ecology, 54, 107–110.

[ece32648-bib-0004] Ancel, L. W. (2000). Undermining the Baldwin expediting effect: Does phenotypic plasticity accelerate evolution? Theoretical Population Biology, 58, 307–319. 11162789 10.1006/tpbi.2000.1484

[ece32648-bib-0005] Armbruster, J. W. (2012). Standardized measurements, landmarks, and meristic counts for cypriniform fishes. Zootaxa, 3586, 8–16.

[ece32648-bib-0006] Banarescu, P. (1992). Zoogeography of fresh waters. Vol. II. Distribution and dispersal of freshwater animals in North America and Eurasia. Wiesbaden, Germany: AULA‐Verlag.

[ece32648-bib-0007] Berner, D. , Adams, D. C. , Grandchamp, A. C. , & Hendry, A. P. (2008). Natural selection drives patterns of lake–stream divergence in stickleback foraging morphology. Journal of Evolutionary Biology, 21, 1653–1665. 18691241 10.1111/j.1420-9101.2008.01583.x

[ece32648-bib-0008] Berner, D. , Grandchamp, A.‐C. , & Hendry, A. P. (2009). Variable progress toward ecological speciation in parapatry: Stickleback across eight lake‐stream transitions. Evolution, 63, 1740–1753. 19228184 10.1111/j.1558-5646.2009.00665.x

[ece32648-bib-0009] Bianco, P. G. (2014). An update on the status of native and exotic freshwater fishes of Italy. Journal of Applied Ichthyology, 30, 62–77.

[ece32648-bib-0010] Bookstein, F. L. (1989). Principal warps: Thin‐plate splines and the decomposition of deformations. IEEE Transactions on Pattern Analysis and Machine Intelligence, 11, 567–585.

[ece32648-bib-0011] Brinsmead, J. , & Fox, M. G. (2002). Morphological variation between lake‐ and stream‐dwelling rock bass and pumpkinseed populations. Journal of Fish Biology, 61, 1619–1638. 10.1111/j.1095-8649.2012.03416.x 23130691

[ece32648-bib-0012] Buchheister, A. , & Wilson, M. (2005). Shrinkage correction and length conversion equations for *Theragra chalcogramma*,* Mallotus villosus* and *Thaleichthys pacificus* . Journal of Fish Biology, 67, 541–548.

[ece32648-bib-0013] Burnaby, T. P. (1966). Growth‐invariant discriminant functions and generalized distances. Biometrics, 22, 96–110.

[ece32648-bib-0014] Cavalcanti, M. J. , Monteiro, L. R. , & Lopes, P. (1999). Landmark‐based morphometric analysis in selected species of serranid fishes (Perciformes: Teleostei). Zoological Studies, 38, 287–294.

[ece32648-bib-0015] Collin, H. , & Fumagalli, L. (2011). Evidence for morphological and adaptive genetic divergence between lake and stream habitats in European minnows (*Phoxinus phoxinus*, Cyprinidae). Molecular Ecology, 20, 4490–4502. 21951706 10.1111/j.1365-294X.2011.05284.x

[ece32648-bib-0016] Collin, H. , & Fumagalli, L. (2015). The role of geography and ecology in shaping repeated patterns of morphological and genetic differentiation between European minnows (*Phoxinus phoxinus*) from the Pyrenees and the Alps. Biological Journal of the Linnean Society, 116, 691–703.

[ece32648-bib-0017] Ehlinger, T. J. , & Wilson, D. S. (1988). Complex foraging polymorphism in bluegill sunfish. Proceedings of the National Academy of Sciences, 85, 1878–1882. 10.1073/pnas.85.6.1878 PMC279884 16578831

[ece32648-bib-0018] Eklöv, P. , & Jonsson, P. (2007). Pike predators induce morphological changes in young perch and roach. Journal of Fish Biology, 70, 155–164.

[ece32648-bib-0019] Frost, W. E. (1943). The natural history of the minnow, *Phoxinus phoxinus* . Journal of Animal Ecology, 12, 139–162.

[ece32648-bib-0020] Garner, P. , Clough, S. , Griffiths, S. , Deans, D. , & Ibbotson, A. (1998). Use of shallow marginal habitat by *Phoxinus phoxinus*: A trade‐off between temperature and food? Journal of Fish Biology, 52, 600–609.

[ece32648-bib-0021] Geiger, M. F. , Herder, F. , Monaghan, M. T. , Almada, V. , Barbieri, R. , Bariche, M. , … Freyhof, J. (2014). Spatial heterogeneity in the Mediterranean Biodiversity Hotspot affects barcoding accuracy of its freshwater fishes. Molecular Ecology Resources, 14, 1210–1221. 24690331 10.1111/1755-0998.12257

[ece32648-bib-0022] Gerstner, C. L. (1999). Maneuverability of four species of coral‐reef fish that differ in body and pectoral‐fin morphology. Canadian Journal of Zoology, 77, 1102–1110.

[ece32648-bib-0023] Ghalambor, C. K. , Hoke, K. L. , Ruell, E. W. , Fischer, E. K. , Reznick, D. N. , & Hughes, K. A. (2015). Non‐adaptive plasticity potentiates rapid adaptive evolution of gene expression in nature. Nature, 525, 372–375. 26331546 10.1038/nature15256

[ece32648-bib-0024] Goatley, C. H. , Bellwood, D. R. , & Bellwood, O. (2010). Fishes on coral reefs: Changing roles over the past 240 million years. Paleobiology, 36, 415–427.

[ece32648-bib-0025] Good, P. (2000). Permutation tests: A practical guide to resampling methods for testing hypotheses. New York, NY: Springer.

[ece32648-bib-0026] Gunz, P. , & Mitteroecker, P. (2013). Semilandmarks: A method for quantifying curves and surfaces. Hystrix, 24, 103–109.

[ece32648-bib-0027] Hendry, A. P. , & Taylor, E. B. (2004). How much of the variation in adaptive divergence can be explained by gene flow? An evaluation using lake‐stream stickleback pairs. Evolution, 58, 2319–2331. 15562693 10.1111/j.0014-3820.2004.tb01606.x

[ece32648-bib-0028] Herler, J. , Lipej, L. , & Makovec, T. (2007). A simple technique for digital imaging of live and preserved small fish specimens. Cybium, 31, 39–44.

[ece32648-bib-0029] Jacquemin, S. J. , Martin, E. , & Pyron, M. (2013). Morphology of bluntnose minnow *Pimephales notatus* (Cyprinidae) covaries with habitat in a central Indiana watershed. The American Midland Naturalist, 169, 137–146.

[ece32648-bib-0030] Kahilainen, K. K. , Siwertsson, A. , Gjelland, K. Ø. , Knudsen, R. , Bøhn, T. , & Amundsen, P.‐A. (2011). The role of gill raker number variability in adaptive radiation of coregonid fish. Evolutionary Ecology, 25, 573–588.

[ece32648-bib-0031] Kerschbaumer, M. , Mitteroecker, P. , & Sturmbauer, C. (2014). Evolution of body shape in sympatric versus non‐sympatric *Tropheus* populations of Lake Tanganyika. Heredity, 112, 89–98. 24065182 10.1038/hdy.2013.78 PMC3907092

[ece32648-bib-0032] Kerschbaumer, M. , Postl, L. , Koch, M. , Wiedl, T. , & Sturmbauer, C. (2010). Morphological distinctness despite large‐scale phenotypic plasticity—Analysis of wild and pond‐bred juveniles of allopatric populations of *Tropheus moorii* . Naturwissenschaften, 98, 125–134. 21161156 10.1007/s00114-010-0751-2 PMC3029815

[ece32648-bib-0033] Klingenberg, C. P. (1998). Heterochrony and allometry: The analysis of evolutionary change in ontogeny. Biological Reviews of the Cambridge Philosophical Society, 73, 79–123. 9569772 10.1017/s000632319800512x

[ece32648-bib-0034] Knebelsberger, T. , Dunz, A. R. , Neumann, D. , & Geiger, M. F. (2015). Molecular diversity of Germany's freshwater fishes and lampreys assessed by DNA barcoding. Molecular Ecology Resources, 15, 562–572. 25186809 10.1111/1755-0998.12322

[ece32648-bib-0035] König, U. , & Borcherding, J. (2012). Preserving young‐of‐the‐year *Perca fluviatilis* in ethanol, formalin, or in a frozen state and the consequences for measuring morphometrics. Journal of Applied Ichthyology, 28, 740–744.

[ece32648-bib-0036] Kottelat, M. (2007). Three new species of *Phoxinus* from Greece and southern France (Teleostei: Cyprinidae). Ichthyological Exploration of Freshwaters, 18, 145–162.

[ece32648-bib-0037] Kottelat, M. , & Freyhof, J. (2007). Handbook of European freshwater fishes. Cornol, Switzerland: Publications Kottelat.

[ece32648-bib-0038] Kristoffersen, J. B. , & Salvanes, A. G. V. (1998). Effects of formaldehyde and ethanol preservation on body and otoliths of *Maurolicus muelleri* and *Benthosema glaciale* . Sarsia, 83, 95–102.

[ece32648-bib-0039] Land, M. , & Nilsson, D.‐E. (2002). Animal eyes. New York, NY: Oxford University Press.

[ece32648-bib-0040] Langerhans, R. B. , Gifford, M. E. , & Everton, O. J. (2007). Ecological speciation in *Gambusia* fishes. Evolution, 61, 2056–2074. 17767582 10.1111/j.1558-5646.2007.00171.x

[ece32648-bib-0041] Langerhans, R. B. , Layman, C. A. , Langerhans, A. K. , & Dewitt, T. J. (2003). Habitat‐associated morphological divergence in two Neotropical fish species. Biological Journal of the Linnean Society, 80, 689–698.

[ece32648-bib-0042] Langerhans, R. B. , & Reznick, D. N. (2010). Ecology and evolution of swimming performance in fishes: Predicting evolution with biomechanics In Domenici P. & Kapoor B. (Eds.), Fish locomotion: An eco‐ethological perspective (pp. 200–248). Boca Raton, FL: CRC Press.

[ece32648-bib-0043] Leinonen, T. , Cano, J. , & Merilä, J. (2011). Genetics of body shape and armour variation in threespine sticklebacks. Journal of Evolutionary Biology, 24, 206–218. 21044205 10.1111/j.1420-9101.2010.02161.x

[ece32648-bib-0044] Lucek, K. , Kristjánsson, B. K. , Skúlason, S. , & Seehausen, O. (2016). Ecosystem size matters: The dimensionality of intralacustrine diversification in Icelandic stickleback is predicted by lake size. Ecology and Evolution, 6, 5256–5272. 27551381 10.1002/ece3.2239 PMC4984502

[ece32648-bib-0045] McGuigan, K. , Franklin, C. E. , Moritz, C. , & Blows, M. W. (2003). Adaptation of rainbow fish to lake and stream habitats. Evolution, 57, 104–118. 12643571 10.1111/j.0014-3820.2003.tb00219.x

[ece32648-bib-0046] McPhail, J. (1993). Ecology and evolution of sympatric sticklebacks (*Gasterosteus*): Origin of the species pairs. Canadian Journal of Zoology, 71, 515–523.

[ece32648-bib-0047] Michel, P. , & Oberdorff, T. (1995). Feeding habits of fourteen European freshwater fish species. Cybium, 19, 5–46.

[ece32648-bib-0048] Mills, C. A. (1987). The life history of the minnow *Phoxinus phoxinus* (L.) in a productive stream. Freshwater Biology, 17, 53–67.

[ece32648-bib-0049] Mills, C. A. , & Eloranta, A. (1985). The biology of *Phoxinus phoxinus* (L.) and other littoral zone fishes in Lake Konnevesi, central Finland. Annales Zoologici Fennici, 22, 1–12.

[ece32648-bib-0050] Mitteroecker, P. , & Bookstein, F. (2011). Linear discrimination, ordination, and the visualization of selection gradients in modern morphometrics. Evolutionary Biology, 38, 100–114.

[ece32648-bib-0051] Mitteroecker, P. , & Gunz, P. (2009). Advances in geometric morphometrics. Evolutionary Biology, 36, 235–247.

[ece32648-bib-0052] Mitteroecker, P. , Gunz, P. , Windhager, S. , & Schaefer, K. (2013). A brief review of shape, form, and allometry in geometric morphometrics, with applications to human facial morphology. Hystrix, 24, 59–66.

[ece32648-bib-0053] Moku, M. , Mori, K. , & Watanabe, Y. (2004). Shrinkage in the body length of myctophid fish (*Diaphus s*lender‐type spp.) larvae with various preservatives. Copeia, 2004, 647–651.

[ece32648-bib-0054] Palandačić, A. , Bravničar, J. , Zupančič, P. , Šanda, R. , & Snoj, A. (2015). Molecular data suggest a multispecies complex of *Phoxinus* (Cyprinidae) in the Western Balkan Peninsula. Molecular Phylogenetics and Evolution, 92, 118–123. 26143109 10.1016/j.ympev.2015.05.024

[ece32648-bib-0055] Palandačić, A. , Ramler, D. , Bravničar, J. , Snoj, A. , & Ahnelt, H. (2015). Resolving phylogeny of the genus Phoxinus in the Western Balkan peninsula with the help auf museum specimens In Fišer Pečnikar Ž. & Lužnik M. (Eds.), Book of abstracts: Biodiversity in the Mediterranean basin (18 pp.). Koper, Slovenia: University of Primorska Press.

[ece32648-bib-0056] Pfennig, D. W. , Wund, M. A. , Snell‐Rood, E. C. , Cruickshank, T. , Schlichting, C. D. , & Moczek, A. P. (2010). Phenotypic plasticity's impacts on diversification and speciation. Trends in Ecology & Evolution, 25, 459–467. 20557976 10.1016/j.tree.2010.05.006

[ece32648-bib-0057] Præbel, K. , Knudsen, R. , Siwertsson, A. , Karhunen, M. , Kahilainen, K. K. , Ovaskainen, O. , … Amundsen, P.‐A. (2013). Ecological speciation in postglacial European whitefish: Rapid adaptive radiations into the littoral, pelagic, and profundal lake habitats. Ecology and Evolution, 3, 4970–4986. 24455129 10.1002/ece3.867 PMC3892361

[ece32648-bib-0058] Price, T. D. , Qvarnström, A. , & Irwin, D. E. (2003). The role of phenotypic plasticity in driving genetic evolution. Proceedings of the Royal Society of London B: Biological Sciences, 270, 1433–1440. 10.1098/rspb.2003.2372 PMC1691402 12965006

[ece32648-bib-0059] Ramler, D. , Delmastro, G. B. , Palandačić, A. , Ahnelt, H. , & Mikschi, E. (2015). Geometric morphometrics as a tool to discover phenotypical divergence In Fišer Pečnikar Ž. & Lužnik M. (Eds.), Book of Abstracts: Biodiversity in the Mediterranean Basin (21 pp.). Koper, Slovenia: University of Primorska Press.

[ece32648-bib-0060] Ramler, D. , Mitteroecker, P. , Shama, L. N. , Wegner, K. , & Ahnelt, H. (2014). Nonlinear effects of temperature on body form and developmental canalization in the threespine stickleback. Journal of Evolutionary Biology, 27, 497–507. 24443968 10.1111/jeb.12311

[ece32648-bib-0061] Robinson, B. W. , & Wilson, D. S. (1994). Character release and displacement in fishes: A neglected literature. The American Naturalist, 144, 596–627.

[ece32648-bib-0062] Robinson, B. W. , & Wilson, D. S. (1995). Experimentally induced morphological diversity in Trinidadian guppies (*Poecilia reticulata*). Copeia, 1995, 294–305.

[ece32648-bib-0063] Rohlf, F. J. (2010a). tpsDig2. Stony Brook, NY: SUNY at Stony Brook, Ecology & Evolution.

[ece32648-bib-0064] Rohlf, F. J. (2010b). tpsRelw. Stony Brook, NY: SUNY at Stony Brook, Ecology & Evolution.

[ece32648-bib-0065] Rohlf, F. J. , & Slice, D. (1990). Extensions of the Procrustes method for the optimal superimposition of landmarks. Systematic Biology, 39, 40–59.

[ece32648-bib-0066] Schluter, D. (2001). Ecology and the origin of species. Trends in Ecology & Evolution, 16, 372–380. 11403870 10.1016/s0169-5347(01)02198-x

[ece32648-bib-0067] Sharpe, D. M. , Räsänen, K. , Berner, D. , & Hendry, A. P. (2008). Genetic and environmental contributions to the morphology of lake and stream stickleback: Implications for gene flow and reproductive isolation. Evolutionary Ecology Research, 10, 849–866.

[ece32648-bib-0068] Simonović, P. D. , Garner, P. , Eastwood, E. A. , Kováč, V. , & Copp, G. H. (1999). Correspondence between ontogenetic shifts in morphology and habitat use in minnow *Phoxinus phoxinus* . Environmental Biology of Fishes, 56, 117–128.

[ece32648-bib-0069] Spoljaric, M. , & Reimchen, T. (2007). 10 000 years later: Evolution of body shape in Haida Gwaii three‐spined stickleback. Journal of Fish Biology, 70, 1484–1503.

[ece32648-bib-0070] Spoljaric, M. , & Reimchen, T. (2011). Habitat‐specific trends in ontogeny of body shape in stickleback from coastal archipelago: Potential for rapid shifts in colonizing populations. Journal of Morphology, 272, 590–597. 21374702 10.1002/jmor.10939

[ece32648-bib-0071] Stearns, S. C. (1982). The role of development in the evolution of life histories In Bonner J. T. (Ed.), Evolution and development (pp. 237–258). Berlin Heidelberg, Germany: Springer.

[ece32648-bib-0072] Straskraba, M. , Chiar, J. , Frank, S. , & Hruska, V. (1966). Contribution to the problem of food competition among the sculpin, minnow and brown‐trout. Journal of Animal Ecology, 35, 303–311.

[ece32648-bib-0073] Svanbäck, R. , & Eklöv, P. (2003). Morphology dependent foraging efficiency in perch: A trade‐off for ecological specialization? Oikos, 102, 273–284.

[ece32648-bib-0074] Svanbäck, R. , & Schluter, D. (2012). Niche specialization influences adaptive phenotypic plasticity in the threespine stickleback. The American Naturalist, 180, 50–59. 10.1086/666000 22673650

[ece32648-bib-0075] Tack, E. (1940). Die Ellritze (*Phoxinus laevis* Ag.), eine monographische Bearbeitung. Archiv für Hydrobiologie, 37, 321–425.

[ece32648-bib-0076] Tamura, K. , Dudley, J. , Nei, M. , & Kumar, S. (2007). MEGA4: Molecular evolutionary genetics analysis (MEGA) software version 4.0. Molecular Biology and Evolution, 24, 1596–1599. 17488738 10.1093/molbev/msm092

[ece32648-bib-0077] Tamura, K. , & Kumar, S. (2002). Evolutionary distance estimation under heterogeneous substitution pattern among lineages. Molecular Biology and Evolution, 19, 1727–1736. 12270899 10.1093/oxfordjournals.molbev.a003995

[ece32648-bib-0078] Tamura, K. , & Nei, M. (1993). Estimation of the number of nucleotide substitutions in the control region of mitochondrial DNA in humans and chimpanzees. Molecular Biology and Evolution, 10, 512–526. 8336541 10.1093/oxfordjournals.molbev.a040023

[ece32648-bib-0079] Tamura, K. , Peterson, D. , Peterson, N. , Stecher, G. , Nei, M. , & Kumar, S. (2011). MEGA5: Molecular evolutionary genetics analysis using maximum likelihood, evolutionary distance, and maximum parsimony methods. Molecular Biology and Evolution, 28, 2731–2739. 21546353 10.1093/molbev/msr121 PMC3203626

[ece32648-bib-0080] Thorstad, E. , Finstad, A. , Jensen, A. , Museth, J. , Naesje, T. , & Saksgård, L. (2007). To what extent does ethanol and freezing preservation cause shrinkage of juvenile Atlantic salmon and European minnow? Fisheries Management and Ecology, 14, 295–298.

[ece32648-bib-0081] Valentin, A. , Penin, X. , Chanut, J. P. , Sévigny, J. M. , & Rohlf, F. (2008). Arching effect on fish body shape in geometric morphometric studies. Journal of Fish Biology, 73, 623–638.

[ece32648-bib-0082] Vega‐Trejo, R. , Zúniga‐Vega, J. J. , & Langerhans, R. B. (2014). Morphological differentiation among populations of *Rhinella marina* (Amphibia: Anura) in Western Mexico. Evolutionary Ecology, 28, 69–88.

[ece32648-bib-0083] Walker, J. A. (1997). Ecological morphology of lacustrine threespine stickleback *Gasterosteus aculeatus* L. (Gasterosteidae) body shape. Biological Journal of the Linnean Society, 61, 3–50.

[ece32648-bib-0084] Wanzenböck, J. , Zaunreiter, M. , Wahl, C. M. , & Noakes, D. L. (1996). Comparison of behavioural and morphological measures of visual resolution during ontogeny of roach (*Rutilus rutilus*) and yellow perch (*Perca flavescens*). Canadian Journal of Fisheries and Aquatic Sciences, 53, 1506–1512.

[ece32648-bib-0085] Ward, R. D. , Zemlak, T. S. , Innes, B. H. , Last, P. R. , & Hebert, P. D. (2005). DNA barcoding Australia's fish species. Philosophical Transactions of the Royal Society of London B: Biological Sciences, 360, 1847–1857. 16214743 10.1098/rstb.2005.1716 PMC1609232

[ece32648-bib-0086] Webb, P. W. (1984). Body form, locomotion and foraging in aquatic vertebrates. American Zoologist, 24, 107–120.

[ece32648-bib-0087] Willacker, J. J. , von Hippel, F. A. , Wilton, P. R. , & Walton, K. M. (2010). Classification of threespine stickleback along the benthic–limnetic axis. Biological Journal of the Linnean Society, 101, 595–608. 21221422 10.1111/j.1095-8312.2010.01531.x PMC3017379

[ece32648-bib-0088] Wootton, R. J. (2009). The Darwinian stickleback *Gasterosteus aculeatus*: A history of evolutionary studies. Journal of Fish Biology, 75, 1919–1942. 20738666 10.1111/j.1095-8649.2009.02412.x

[ece32648-bib-0089] Zardoya, R. , & Doadrio, I. (1999). Molecular evidence on the evolutionary and biogeographical patterns of European cyprinids. Journal of Molecular Evolution, 49, 227–237. 10441674 10.1007/pl00006545

