# Supplementary material for: Morphological divergence of lake and stream Phoxinus of Northern Italy and the Danube basin based on geometric morphometric analysis
Source: Ecol Evol. 2016 Dec 20;7(2):572–84. doi: 10.1002/ece3.2648 (PMC5243779; doi:10.1002/ece3.2648)
Supplement: Supplementary file 1 [file ECE3-7-572-s001.docx]

Table S1. Comparison of morphological characters on lateral, ventral, and dorsal body side of minnow populations from Northern Italy and the Danube basin. ITA L, ITA S: Italian lake and stream populations. DAN L, DAN S: Danubian lake and stream populations.

| Groups | ITA L vs. ITA S | DAN S vs. ITA S | DAN L vs. ITA L |
| --- | --- | --- | --- |
| Lateral | - slender bodies  - narrower caudal peduncles  - shorter base of anal and dorsal fin  - larger head  - larger eyes  - terminal mouth vs. subterminal mouth  - shape variance lower | - slender bodies  - narrower and longer caudal peduncles  - shape variance higher | - slender bodies  - narrower and longer caudal peduncles  - larger eyes  - higher shape variance |
| Ventral | - slender body and narrower gape  - bases of pectoral fins closer together  - base of pectoral fin longer and more  vertically positioned  - both high shape variances, in ITA L  higher | - slender body and shorter and wider gape  - bases of pectoral fins closer together  - base of pectoral fin longer and more  horizontally positioned  - distance between pectoral and pelvic fins  shorter  - distance between anus and anal fin longer  - both high shape variances, in ITA S higher | - slender body and shorter and broader gape  - bases of the pectoral fins further apart  - pelvic fins closer together and their bases  shorter  - distance between pectoral and pelvic fins  shorter  - distance between anus and anal fin longer  - both high shape variances, in ITA L higher |
| Dorsal | - not significant | - slightly longer and broader head  - head to dorsal fin distance shorter  - shape variances lowest on this side | - slightly longer and broader gape  - head to dorsal fin distance shorter  - shape variances lowest on this side |
